# Joy Leads to Overconfidence, and a Simple Countermeasure

**DOI:** 10.1371/journal.pone.0143263

**Published:** 2015-12-17

**Authors:** Philipp Koellinger, Theresa Treffers

**Affiliations:** 1 Erasmus School of Economics, Erasmus University Rotterdam, Rotterdam, The Netherlands; 2 Faculty of Economics and Business, University of Amsterdam, Amsterdam, The Netherlands; 3 Department of Complex Trait Genetics, Center for Neurogenomics and Cognitive Research, Vrije Universiteit Amsterdam, Amsterdam, The Netherlands; 4 School of Industrial Engineering & Innovation Sciences, Eindhoven University of Technology, Eindhoven, The Netherlands; Middlesex University London, UNITED KINGDOM

## Abstract

Overconfidence has been identified as a source of suboptimal decision making in many real-life domains, with often far-reaching consequences. This study identifies a mechanism that can cause overconfidence and demonstrates a simple, effective countermeasure in an incentive-compatible experimental study. We observed that joy induced overconfidence if the reason for joy (an unexpected gift) was unrelated to the judgment task and if participants were not made specifically aware of this mood manipulation. In contrast, we observed well-calibrated judgments among participants in a control group who were in their resting mood. Furthermore, we found well-calibrated judgments among participants who received the joyful mood induction together with questions that forced them to reflect on their current mood and the (ir)relevance of its cause to our judgment tasks. Our findings suggest that being aware of one’s positive mood and the reason for that mood may effectively reduce overconfidence for a short period.

## Introduction

Overconfidence is “widespread, stubborn, and costly” ([1], p. 257). For example, entrepreneurs are often overconfident about their chances of success and their own skills compared to others, which often results in suboptimal returns on their investments (e.g., [2–5]). Financial investors, both amateur and professional, “stubbornly believe that they can do better than the market, contrary to an economic theory that most of them accept and contrary to what they could learn from a dispassionate evaluation of their personal experience” ([1], p. 217, [6, 7]). Bettors who gamble on football games prefer low-probability wagers—and frequently lose—because they have too much confidence in their own expertise [8]. Inventors also appear to be more overconfident and optimistic compared with the general population. They often continue to invest substantial amounts of time and money into their ideas despite strongly supported advice to cease their efforts—not surprisingly, the end results are mostly disappointing [9, 10]. Managers, planners, and even academics often ignore statistical information about how long it will take them to complete large projects. Instead, they rely on their own expertise and insight, which often results in unrealistic plans, unexpected delays, higher costs and disappointing outcomes [11, 12].

Overconfident CEOs tend to overestimate the returns on their investment projects and fail to reduce their personal exposure to company-specific risk [13]. They frequently overpay for target companies and undertake value-destroying mergers [14]. Nevertheless, overconfidence may help managers become promoted to CEO in the first place [15].

Examples of overconfidence are not just limited to the economic and social science spheres: Medical studies have shown that physicians’ overconfidence is likely to be an important reason for severe diagnostic errors that result in increased patient morbidity and mortality [16]. In 2008, the *American Journal of Medicine* devoted an entire special issue to overconfidence and clinical decision making (see Issue 5) and cited overconfidence as one of the most common reasons for medical errors among physicians.

In some cases, overconfidence is even a matter of war and peace: Rational states should not go to war against one another without thinking they can win—and clearly, at least one side must be wrong [17]. The ability to avoid bad judgments and decisions that are induced by overconfidence is therefore important (e.g., [18, 19, 20, 21]).

However, our understanding about where overconfidence comes from or how it can be reduced is limited. Previous studies have suggested that overconfidence is influenced by gender differences (e.g., [6, 22]) and differences in personality [23]. Furthermore, twin studies show a moderate heritability of overconfidence [24], suggesting that some people are more prone to overconfidence for biological reasons. In line with the biological argument, the ratio of the second to fourth finger (2*D*:4*D*, considered to be a marker of early exposure to testosterone) was found to be related to men’s overconfidence [25]. Similarly, 2*D*:4*D* was shown to be related to risk-taking and abstract reasoning ability [26]. However, gender, personality, heritability, and 2*D*:4*D* estimates neither explain the underlying temporary mechanisms that may lead to overconfidence nor do they suggest potential ways of reducing it.

The present study addresses this gap and takes a novel approach toward investigating affect as a transient cause for overconfidence and testing the effectiveness of a potential, temporary countermeasure. Preliminary advances have been made in the study of affect and overconfidence. For example, [27] studied the influence of mood and framing on overconfidence. Although they did report significant interaction effects of mood and framing on overconfidence, they did not find direct effects of mood on overconfidence. In addition, [28] and [29] also reported that modest levels of elated and sad mood do not influence confidence judgments. These insignificant effects may be explained by the fact that these studies examined the effects of positive affect compared to those of negative affect instead of comparing the effects of positive affect to those of neutral affect. Comparing neutral to positive affect, a recent study by [30] found that mild positive affect increases overconfidence. We attempt to go a step further in our study by seeking a potential mechanism for this result that suggests a possible debiasing strategy.

Furthermore, in this study, we identify affect-as-information as a causal mechanism for the influence of positive affect on overconfidence. Building on affect-as-information theory [31], we show that questioning the informational value of one’s affective state reduces overconfidence. Although studies from several fields such as social psychology [32, 33, 34], consumer psychology (e.g., [35, 36]), and finance [37] have shown that this mechanism applies to various judgments, to the best of our knowledge this is the first study demonstrating the effectiveness of this mechanism for overconfidence. Hence, we contribute to existing literature by examining the transient causal effect of joy—a distinct positive affective state—on overconfidence and show that joy’s effects on overconfidence can be reduced when calling the informational value of individuals’ affective state into question.

### Overconfidence

Overconfidence is a bias in beliefs that induces deviation from payoff-maximizing behavior [38]. People who are imprecise in the judgment of their own skills and abilities are defined as *absolutely* over- or underconfident [39, 40]. People who are biased in their judgment of themselves relative to others are defined as *relatively* over- or underconfident [41, 42]. The distinction between judgments of absolute and relative performance is important because self-reported judgments are psychometrically different from comparative judgments [43], and the mental processes that lead to these judgments may differ [44]. Therefore, we investigate the influence of joy on *absolute* and *relative* overconfidence in one’s own performance on a general-knowledge task.

Previous studies have shown that overconfidence is a context- and task-dependent phenomenon. For example, absolute overconfidence appears to be greatest during difficult tasks, whereas relative overconfidence appears to be greatest during easy tasks [41, 42, 45, 46]. These inconsistencies may be explained by the fact that knowledge about one’s own performance is uncertain, whereas knowledge about the performance of others is even more uncertain. Consequently, people’s post-task self-assessments are regressive, and their estimates of others are even more regressive [46]. However, these insights do not help people avoid overconfidence during a given decision-making situation.

### Potential Debiasing Strategies against Overconfidence

[18] reviewed several methods that may be used in specific situations to avoid overconfidence or at least limit its damage-causing potential. For example, the authors suggest that engineers calculating the amount of concrete needed to hold a dam or a bridge may benefit from multiplying their calculations by a safety factor of 3 or even 8. Similarly, managers may add “buffer time” into their budgets when they plan projects. However, we could not find published empirical results that probe the effectiveness of these ideas.

Another possible debiasing strategy against overconfidence may be to “think of the opposite” by articulating reasons why an initial answer might be wrong or why a project idea might fail [47]. Modified versions of this debiasing strategy have been discussed by Gary Klein ([1], pp. 255–265) as “premortem” and by [48] as “prospective hindsight.” For example, the idea of the “premortem” is that an individual or a group of people responsible for making a particular decision should meet just before making the final commitment to a project. Decision makers stage a hypothetical event one year into the future in which they discuss why the project has miserably failed. The possible effectiveness of contemplating a past failure rests on unleashing the imagination of knowledgeable individuals and on legitimizing doubts about the project. Although we could not find empirical evidence that this debiasing strategy works, it may in principle dampen excessive optimism.

Some empirical evidence suggests that skeptical feedback from knowledgeable persons who are not personally involved in a project can help prevent overconfidence to some extent [49, 50]. More generally, confronting people with objective risk information may reduce the bias in their judgments [51]. [12] argued that overconfidence may be prevented by focusing on an “outside view” that looks beyond the details of the specific situation and instead examines statistical information about a group of cases that are similar in relevant respects. For example, an aspiring restaurant owner should not ask how likely it is that her particular business will succeed. Instead, she should ask what the average survival rate and financial performance of all restaurants in the relevant area have been in the past few years. This outside perspective could provide some protection against forecasts that are not within the realm of reasonable possibilities. However, objective information about statistical risk is often difficult to obtain, and in many cases, it is questionable what the relevant reference category for a specific situation should be. This limitation is particularly relevant for situations that involve a high degree of novelty [18].

In addition, an early study by [52] reported that feedback on results from a general knowledge task significantly reduced overconfidence. In contrast, [53] also tested immediate feedback of results in a general knowledge task as a potential countermeasure for overconfidence but did not find consistent significant effects of external feedback to reduce overconfidence. [54] also showed that giving participants feedback about their results could not de-bias judgments of learnings.

A judgment bias related to overconfidence is overprecision that describes individuals who are inaccurate in the precision of their judgment (e.g., [7]). A recent review by [55] about general de-biasing strategies reported several effective countermeasures against overprecision. For example, participants who were forced to split questions into multiple parts improved their performance through intervals that were wider and better centered [56]. In addition, people who were urged to consider all possible outcomes of an event show reduced overprecision [57]; this strategy has also been shown to effectively reduce overprecision in order behavior [58]. Finally, another study by [59] showed that actively open-minded thinkers are more likely to make accurate judgments.

In the present study, we examine absolute and relative overconfidence of individuals. The decision-making situation we investigate in the present study resembles that faced by individuals who need to make accurate judgments about their own performance without feedback or any other outside information that they could use to anchor their beliefs. Hence, the debiasing strategies against overconfidence discussed above are not available to our decisions makers. Instead, their only source of information is their own knowledge and experiences and the experimental environment. In such situations, people’s confidence judgment may be influenced by their moods and emotions. Indeed, extensive literature has identified the crucial influence of affective states on judgment and on decision making more generally (e.g., [31, 60–62]), and some scholars have also started investigating these relationships in the brain (e.g., [63]). We refer to this literature and, as one of the first studies in this area, link it to overconfidence.

### Affect and judgment

Affect is often used as an umbrella term for moods and emotions. Whereas moods can be defined as diffuse, objectless affective states, emotions are regarded as affective states that are typically directed toward a certain object and that manifest in particular action tendencies, expressive reactions, and physiological changes [64]. In this study, we use the term “mood” instead of “emotion” because we believe that the definition of mood fits our study design better than the definition of emotion does.

We apply a discrete view on affective states by focusing on joy as one of humans’ core affective states [65, 66]. We distinguish joy from happiness because happiness is often defined as the frequency, not the intensity, of experiencing positive affect [67]. Hence, joy is more suitable to describe a short-lived affective state as we study it in this research. In addition, we focus on joy as a basic positive affect because joy is the only positive affective state in the discrete view. In contrast, the discrete view distinguishes between several negative states, such as fear, anger, or sadness. However, negative affects do not necessarily have opposing effects to positive affects because negative affects are more distinct among themselves than positive affects. Instead, the effect of positive and negative affects should instead be compared to neutral affect for easier interpretability [68]. For example, comparing the effects of joy to the effects of sadness or to the effects of anger may be misleading in identifying the incremental effects of joy. Indeed, in comparing positive and negative affects, studies investigating the influence of affect on overconfidence have not found significant effects [28, 29]. Their non-findings could, however, also be related to other aspects of their study design. Nevertheless, a recent study by [69] found that mild negative affect—compared to neutral affect—can increase overconfidence.

Negative and positive affective states have been shown to directly [31] or indirectly [70] influence judgments in an affect-congruent manner. Typically, people’s resting mood is slightly positive [31], and the cause of this base-level positive mood is typically diffuse and not salient to the individual [60]. Studies that do not make the cause of a given mood salient typically report affect-congruent judgments in various contexts, such as life satisfaction [31], consumer products [71], culpability [37], risk estimates [72], and estimates of physical space [73].

Affect-congruent effect on judgments can be explained by the concept-priming hypothesis [70, 74] and the affect-as-information hypothesis [31]. Although these hypotheses present different, but possibly parallel, mechanisms for how affect influences judgments, both state that current affective states result in affect-congruent outcomes.

The concept-priming hypothesis suggests that affect may prime (i.e., activate in memory) material that is affect-congruent, leading to affect-congruent judgments [70]. Happy moods may facilitate the recall of happy memories and inhibit the recall of sad memories, and vice versa [74]. These affect-congruent memories result in affect-congruent judgments and decision outcomes.

In contrast, the affect-as-information hypothesis proposes that affective cues of moods and emotions directly influence judgments by providing experiential and bodily information that is attributed to the object of judgment [31, 60]. Thus, individuals may ask themselves, “How do I feel about it?” In doing so, they may mistake pre-existing feelings for a reaction to the target. Therefore, positive affect could encourage individuals to continue, thereby providing them with a “go” signal leading to affect-congruent judgments, such as an increase in confidence.

Building on the affect-as-information hypothesis, studies have shown that the misattribution of irrelevant causes of affective states to judgment objects that leads to affect-congruent judgments may weaken or disappear entirely if the irrelevant source of the affect is salient (e.g., [31–37]). A preliminary study by [31] found that the effect of negative moods on life satisfaction and happiness was eliminated when the irrelevant source of the affective information (i.e., the weather) was made explicit. When participants were provided with an explanation for their current negative affective state that was irrelevant to their evaluation of their lives, they were less likely to use their affective cues as an informational basis for their evaluative judgments. Subsequent studies have also shown this debiasing effect for positive and negative affects on other judgments such as product evaluations [35, 36], risk [33], or evaluations of others [34]. Hence, based on these previous studies, we hypothesize that people also use their positive affect, such as joy, as (irrelevant) information that induces overconfidence. Similarly, we hypothesize that questioning joy’s informational value may reduce people’s tendency to be overconfident when they are in a positive mood. To the best of our knowledge, no studies have yet investigated these affect-as-information inspired hypotheses in the context of overconfidence.

### The Present Research

Our study examines these hypotheses in a carefully controlled, randomized laboratory experiment. To measure overconfidence, we employed a general knowledge task of medium difficulty that can be expected to have a relatively high number of well-calibrated judgments compared with those afforded by very easy or very difficult tasks [41, 42, 45, 46]. Although this task decreases our a priori chance of observing overconfidence and places an upper boundary on the possible effect sizes of the countermeasure against overconfidence that we study, we believe that most of the relevant decision-making situations we named above fall into the medium difficulty category, which also makes our study comparable to that of [30] and our own previous work (see S1 Text). Furthermore, we use an incentive-compatible method [75] to elicit confidence judgments that further improve internal and external validity because the precision of judgments has been shown to depend on whether the decision-maker faces real (financial) consequences [39, 45, 76].

## Materials and Methods

### Data availability

The data and the full set of experimental instructions from this study can be found at https://dataverse.harvard.edu/

### Prior considerations

To aid the design of our experiment, we attempted to gauge the plausible effect sizes of experimental mood manipulations on overconfidence by consulting prior literature and by conducting an exploratory pretest at a large public university in Germany (see S1 Text). The study by [30] was the only existing study we could identify that induced positive affect and measured the effect of this mood manipulation on overconfidence. We computed the effect sizes of the experimental treatment on absolute and relative overconfidence in [30] using the reported regression coefficients and their standard errors. The positive mood induction had direct effects that varied between *Cohen’s d* = 0.22 and *d* = 0.62, depending on the model specification. Cohen’s *d* is a standardized effect size for the comparison of two means in terms of the standard deviation (*SD*) of the data. For example, if the variable of interest has *SD* = 15 in the pooled sample and one sub-group has a mean that is 7.5 higher than the other sub-group, then *d* = 0.50. [77] suggests that *d* = 0.20 describes a small effect, *d* = 0.50 a medium effect, and *d* = 0.80 a large effect. The effects of the positive mood induction on absolute and relative overconfidence in our own pretest varied between *d* = 0.28 and *d* = 0.63, also depending on the model specification. The sample-size weighted average effect size observed in these studies was *d* = 0.42 (please see Table 1 for an overview of the effect sizes in prior studies).

**Table 1:** Effect sizes in prior studies.

Source	Dependent variable	Control variables	*β*	*SE(β)*	*t* statistic	Cohen’s *d*	*N*
[28]	*OC*	No	0.94	0.81	1.16	0.22	107
		Yes	2.34	0.46	3.09	0.62	99
	*ROC*	No	1.04	0.88	1.12	0.22	104
		Yes	1.28	0.48	1.99	0.39	98
Pretest	*OC*	No	0.06	0.05	1.20	0.33	52
		Yes	0.05	0.05	1.00	0.28	52
	*ROC*	No	0.25	0.11	2.27	0.63	52
		Yes	0.16	0.07	2.29	0.63	52

*Note*: *OC* stands for absolute overconfidence, *ROC* for relative overconfidence. *β* is the unstandardized regression coefficient comparing the effect of the *joy* treatment group with that of the *control* group. *β*’s from [30] are taken from Tables 4 and 5, models 1 and 3. Their control variables included sex, proportion of females in the session, demographic controls, disgust, embarrassment, and performance on a quiz (number of correct responses). The control variables in the analyses from our pretest included sex, age, age^2^, financial stakes, study subject, and performance (percentage of correctly answered quiz questions). Excluding performance as a control variable yielded slightly higher estimated effects of the experimental treatment in our pretest. We do not know what the results of [30] would look like if performance were excluded as a control variable. The *t* statistic is calculated as *β* / *SE(β)*. Cohen’s *d* was calculated using *t* and *N*. The standard errors of Cohen’s *d*s in [30] were 0.20; standard errors of Cohen’s *d*s in our pretest were 0.29.

Our own pretest and the study by [30] induced joy using film clips combined with questionnaires about the participants’ moods that were administered either before and after the overconfidence measure (in our pretest) or only after the measure (in [30]). The effects of the joy induction on both overconfidence measures were diametrically opposed in these two studies. This result and theoretical considerations based on extant literature led us to hypothesize that the influence of positive affect on overconfidence critically depends on whether the irrelevant source of positive affect is salient to the participants. Participants completing a mood manipulation check after the joy induction and before the task were very likely to be aware of the informational value of their affective state. Therefore, we refrained from applying mood manipulation checks in the main study to avoid this redeeming effect. Instead, we designed our main study to specifically test this assumption. We expected somewhat larger effect sizes within our main experiment (E(*d*) ≈ 0.5) than in the previous studies because we aimed to isolate the effect that we thought caused the large differences between the results of our own pretest and those of the study by [30].

Based on our expected effect size of *d* ≈ 0.5 and practical considerations, we decided at the outset to terminate data collection after one week in the laboratory, with the aim of recruiting at least 50 participants for each of four experimental groups. A priori power calculations showed that with *N*_group_ = 50 and *d* ≈ 0.5, we would have 80% power to find between-group differences in means with *p* = 0.05 in a one-sided test (using G*Power 3.1.7) [78]. We conducted our main experiment in a different university and country from our pretest and the study by [30] to ensure the independence of observations. Data analyses started after data collection was finished.

### Participants

Our main experiment was conducted at a large public university in the Netherlands. The Ethics Committee from the Erasmus University Rotterdam in the Netherlands approved our main experiment, and all participants provided written informed consent. The study included 226 participants who were randomly distributed across four groups. Because of the large pool of 2,500 participants from which we recruited, most of the participants did not know any other participants in the experiment. We conducted analyses of variances (ANOVA), and neither the date nor the time of the experimental sessions affected participants’ absolute (date: *F*[4, 203] = 1.37, *p* = 0.25; time: *F*[4, 203] = 0.83, *p* = 0.53) or relative (date: *F*[4, 203] = 0.09, *p* = 0.99; *F*[5, 203] = 1.50, *p* = 0.19) overconfidence.

The sample consisted of 124 male and 102 female management and economics students with an average age of 21 years (*SE* = 0.16). The youngest and oldest participants were 18 and 29 years old, respectively. The participants were informed that they would participate in an experiment on decision making that had real financial payoffs. They completed the experiment in a cubicle without the ability to communicate with other participants, and there were no time restrictions for completing the experiment (average completion time was 30 minutes).

### Procedure and experimental treatments

For every session, participants drew a number upon arrival that randomly assigned them to a cubicle in the laboratory. The participants signed consent forms confirming that they had read and understood the terms and conditions of the experiment and that the experimenters had adequately answered all of their questions. In every session, we had participants in the control group and all three treatment groups. We conducted a total of 30 sessions in one week with 6 sessions per day and up to eight participants per session. All experimental sessions lasted less than one hour (please see Table 2 for the experimental design).

**Table 2:** Experimental Design.

	Before experiment	Experiment			*N*
**Control**	-		O_1_	O_2_	***51 (55)***
**Joy treatment**	X_1_		O_1_	O_2_	***53 (58)***
**Joy awareness**	X_1_	X_2_	O_1_	O_2_	***55 (56)***
**Overconfidence**		X_3_	O_1_	O_2_	***54 (57)***

*Note*. *N* = 213 (226). The numbers in brackets include participants who did not understand the experimental task, made unreasonable choices, or had computer problems. We excluded these 13 participants from further analyses, but our results are robust to the inclusion of these participants. X_1_ = joyful affect induction (i.e., gift), X_2_ = joy awareness treatment (i.e., scale), X_3_ = overconfidence awareness treatment (i.e., text), O_1_ = overconfidence task, O_2_ = standard questions.

Before the experiment started, half of the participants received a small bag of gummy bears as an unexpected gift (*X*_1_). The gummy bears were always handed out by the same experimenter, who entered the cubicles with a smile and the words, “This is a thank-you gift for your participation. The experiment will start in a few minutes.” The gifts were distributed so that the other participants did not notice. Gift-related mood induction processes have been regularly and successfully used to induce positive mood states [62, 79, 80, 81]. Based on the evidence from these previous studies and for the purpose of this treatment, we refrained from applying manipulation checks after we handed out the gift. We used the gift for two different purposes, which resulted in two different treatment groups. First, we used the gift to amplify participants’ positive resting mood, without making the purpose of this intervention obvious. This treatment represents the *joy* group.

Second, we used the gift to provide participants in the *joy awareness* group with a salient cause for their joyful mood that was clearly irrelevant to the judgment tasks that would follow. To make the irrelevant source (i.e., the gift) of this mood manipulation evident, participants in the *joy awareness* group answered a short scale (*X*_2_) after they received the gummy bears and before the experiment began. The participants indicated their answers to four short statements on a 10-point Likert-type scale with the poles 1 “I strongly disagree” and 10 “I strongly agree” [32, 33]. The statements were as follows: “1. I am currently in a joyful mood” (*Mean* = 7.6, *SE* = 0.20); “2. The present I received for participating in this experiment had a positive influence on my mood” (*Mean* = 8.0, *SE* = 0.25); “3. The fact that I received a present does not influence how much I know about science and culture” (*Mean* = 8.9, *SE* = 0.27); and “4. The fact that I received a present should not influence my ability to make good judgments” (*Mean* = 8.5, *SE* = 0.30). Responses to the first two items indicate that the gift had a positive effect on participants’ mood; responses to the second two items link the gift, which increased participants’ positive mood, as an (irrelevant) source to the quality of their judgment.

One-quarter of the participants received a treatment that made them aware of the influence of overconfidence on people’s decisions at the beginning of the experiment, without receiving a gift. Our *overconfidence awareness* group received a treatment in the form of a short text (*X*_3_) stating that overconfidence is a widespread and notorious bias that negatively influences most people’s judgments and decisions. The text further stated that participants could maximize their payoff in this experiment if they were aware of this bias and if they judged their performance accurately (please see S2 Text for details about experimental design). We included this treatment because the control group in our pretest was clearly overconfident, and we were interested in determining whether informing people about the overconfidence bias would also lead to better-calibrated judgments.

The *control* group in our main experiment did not receive a gift or a particular pretext but started directly on the overconfidence measure (*O*_1_). For all other groups, *O*_1_ followed the experimental treatment(s). The instructions for the experimental task (please see below and S2 Text, details about experimental design) stated that the participants’ payoff at the end of the experiment would depend on their performance on the subsequent task. The participants received an attendance fee of 4 EUR and the opportunity to earn up to 12 EUR more.

After the experimental task, participants answered standard questions (*O*_2_) about their age, gender, current occupation, and level of education. The participants also completed short measures of personality (e.g., “I see myself as extraverted, enthusiastic,” “I see myself as critical, quarrelsome”) [82], risk preferences (“I am generally a person who is fully prepared to take risks”) [83], general self-efficacy (e.g., “I can always manage to solve difficult problems if I try hard enough,” “If someone opposes me, I can find the means and ways to get what I want;” *Cronbach’s α* = 0.81) [84], and optimism (e.g., “In uncertain times, I usually expect the best,” “I hardly ever expect things to go my way;” *α* = 0.72) [85]. Table 2 presents an overview of the experimental design, which was adapted and extended from the pretest.

Internal reliabilities for measurements of extraversion (Cronbach’s *α* = 0.63), agreeableness (*α* = 0.01), conscientiousness (*α* = 0.60), emotional stability (*α* = 0.69), and openness (*α* = 0.41) were not satisfactory. Hence, we used the ten personality items for a rotated factor analysis and extracted four components with eigenvalues that were greater than 1, namely, extraversion, emotional stability, conscientiousness, and openness. Agreeableness was not found to be a separate factor and was therefore not included in the analyses. The principal component analysis (PCA) considers all of the available information, whereas the sum scores of the items for one personality dimension ignore the personality differences among participants. The factor scores and sum scores were almost perfectly correlated for all four of the five examined personality dimensions (*r* > 0.88, *p* < 0.001).

At the end of the experiment, payoffs were determined by the computer according to the experimental instructions and observed behavior. Participants received their payoffs separately and sequentially after they signed individual receipts. On average, participants earned 7.20 EUR (*SE* = 0.29); 34 participants earned nothing above their participation fee, 98 participants earned an additional 6 EUR, and 81 participants received 12 EUR in addition to their participation fee.

### Overconfidence Measure

To elicit the participants’ beliefs about their absolute and relative performance, we used the incentive-compatible experimental design developed by [44] and a general knowledge quiz with ten multiple-choice questions of medium difficulty, e.g., “Earth equator is around … 000 km long,” “First Tour de France took place in year …” [39]. In the method reported by [44], the participants’ payoffs depended on their absolute performance on the general knowledge quiz and their choices between two different payoff mechanisms. Specifically, each participant completed two sets of ten binary payoff choices. One set elicited absolute confidence, and the other set elicited relative confidence. We randomized the order of these sets to prevent ordering effects.

In both sets of binary choices, the participants decided which of two payoff mechanisms they preferred. One of the two payoff mechanisms was a random lottery with a known probability of winning. The probabilities of winning increased in ten steps from 5% to 95% from choice one to ten for both sets. The alternative payoff mechanism depended on how many questions the participants answered correctly in the general knowledge quiz. In the measure of absolute overconfidence, this payoff mechanism generated a win if one randomly chosen quiz question was answered correctly. In the measure of relative overconfidence, the payoff mechanism generated a win if another randomly chosen participant in the experiment answered *fewer* questions correctly. In the case of a draw between both participants, a fair coin flip decided the winner. For every set of choices, participants could win 6 EUR.

The participants did not receive feedback about their performance on the quiz. Therefore, they had to exercise careful judgment about their performance to choose the payoff mechanism that maximized their expected payoff. The switching point from the performance-based payoff mechanism to the lotteries with known probabilities was used to compute the levels of absolute and relative overconfidence.

This method has a number of important advantages over existing methods. First, rather than directly asking participants for their level of confidence, their beliefs are elicited in a strictly incentive-compatible experiment [75] that strongly reinforces deliberate behavior and precise judgment (see [44] for the proof). This measure is important because appropriate financial incentives improve the precision of a judgment [45, 76, 86] and influence behavior in risky situations [87, 88]. Second, the measure allows for absolute and relative overconfidence to be differentiated within subjects. In our experiment, absolute overconfidence refers to overestimating the number of correctly answered quiz questions, whereas relative overconfidence refers to overestimating one’s own performance in answering the quiz questions compared to a randomly chosen opponent. Third, the measure differentiates between people with well-calibrated judgments and those without and allows the degrees of overconfidence to be specified. Fourth, the measure reported by [44] is robust to risk preferences as long as subjects (1) have source-independent risk attitudes (as assumed by prospect theory or cumulative prospect theory). Intuitively, subjects are choosing between two payoff mechanisms that resemble lotteries: In one lottery, the source of risk is a random device. In the other lottery, the source is one’s own performance (in absolute terms or relative to someone else). The measure systematically varies the probability of the random device and elicits beliefs about own performance by evaluating subjects’ switching point between the two lotteries. Thus, any preference for probability distributions is orthogonal to the observed switching point as long as subjects do not prefer one source of uncertainty over another. Finally, the method provides unambiguous monetary incentives for participants to maximize the number of correctly answered quiz questions because their chance of winning strictly increases with their performance in the quiz. This feature is important because we are interested in examining the participants’ ability to judge their own maximum performance, not their ability to optimize the predictability of their performance [46, 89].

Based on these data, six measurements were generated (see [46]). *Absolute performance* (*ap*) was measured as the percentage of correctly answered quiz questions. *Relative performance* (*rp*) took a value of 1 if the participant performed better than a randomly assigned participant, 0 otherwise, and 0.5 in the case of a draw between the two participants. *Confidence* (*c*) was measured by the switching point from the payoff mechanism based on *absolute* performance to the lottery with known probabilities. For example, the case in which a participant chooses the absolute performance payoff when the alternative is winning with 45% or less but otherwise chooses the lotteries that have a probability of winning 55% or more implies that the participant believes that he or she answered 50% of the quiz questions correctly (*c* = 0.5). *Relative confidence* (*rc*) was measured by the switching point from the payoff mechanism based on *relative* performance to the lottery with known probabilities. For example, the case in which a participant chooses the relative performance payoff when the alternative is winning with 65% or less but otherwise chooses the lotteries that have a 75% or higher probability of winning implies that the participant believes that his or her performance ranks in the top 30% of all people who participated in the experiment (*rc* = 0.7). *Absolute overconfidence* (*oc*) is the difference between absolute confidence and absolute performance, *oc = c−p*. *Relative overconfidence* (*roc*) is the difference between relative confidence and relative performance, *roc = rp−rc*. Both *oc* and *roc* can vary between -1 and 1, where a value of -1 indicates underconfidence, 0 indicates a well-calibrated judgment, and 1 indicates overconfidence.

We calculated *roc* by comparing with the median performance in the experiment rather than a randomly chosen opponent. This procedure removes the noise resulting from the random matching of participants from the relative overconfidence measure *roc*. The random matching procedure implies that a participant would sometimes be classified as over- or underconfident depending on the randomly assigned opponent. Because the participants could not guess who their opponent would be, the best they could do was to form an opinion about how good the median participant in this experiment would be and whether they believed themselves better than the median. Nevertheless, we conducted additional tests to evaluate the robustness of our findings to various outcomes of random matches among participants.

## Results

On average, participants answered 4.6 (*SE* = 0.13) out of 10 questions correctly. The median performer answered five questions correctly. The best and the worst performances were 10 (*N*_*10*_ = 2) and 0 (*N*_*0*_ = 1) questions answered correctly, respectively. An analysis of variance (ANOVA) revealed no significant differences between the experimental groups in their performance (*F*[3, 209] = 0.32, *p* = 0.81, *partial η* ^*2*^ = 0.01) or their performance range (test of homogeneity of variances, Levene’s *F*(3, 209) = 0.57, *p* = 0.63). An analysis of variance (ANOVA) is the same method as a multivariate linear regression, but only presents the results differently. In an ANOVA, each experimental group’s mean is compared to the grand mean. In a linear regression, the experimental group variable is dummy coded, which means that each experimental group’s intercept is compared to the reference group’s intercept. Since the intercept in a linear regression is defined as the mean value when all other predictors are zero, and there are no other predictors, the three intercepts are just means. Hence, an ANOVA reports each mean and a p-value that reports whether at least two of the means are significantly different (i.e., an omnibus test); ANOVA post-hoc tests can show which of the means are significantly different. A regression analysis reports only one mean (as an intercept), and the differences between that one and all other means, but the p-values evaluate those specific comparisons. *Partial eta squared* (*partial η* ^*2*^) describes the proportion of variability associated with an effect when the variability associated with all other effects identified in the analysis has been removed from consideration [90]. [77] describes *partial η* ^*2*^ = 0.01 as a small effect, *partial η* ^*2*^ = 0.06 as a medium effect, and *partial η* ^*2*^ = 0.14 as a large effect.

**Fig 1:**
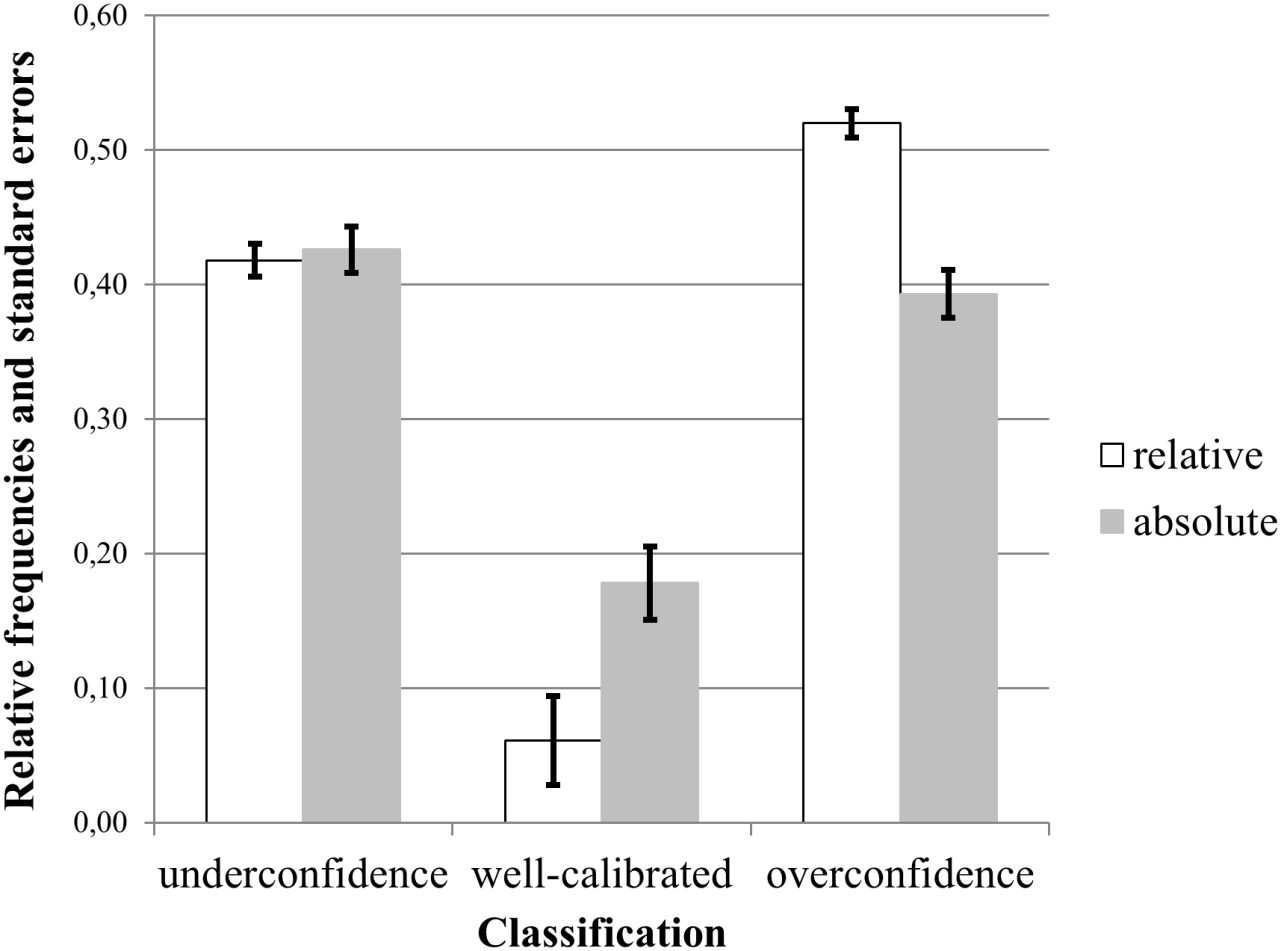
The relative frequency of under- and overconfident judgments for absolute (ap) and relative (rp) performance and their standard errors (*N* = 213).

Fig 1 shows the distribution of well-calibrated, under- and overconfident participants in our sample. On average, self-confidence judgments were better calibrated for absolute (*ap*_*well-calibrated*_ = 0.18, *SE* = 0.03) than for relative (*rp*_*well-calibrated*_ = 0.06, *SE* = 0.03) performance. Underconfidence occurred equally for absolute (*ap*_underconfidence_ = 0.43, *SE* = 0.04) and relative performance (*rp*_*underconfidence*_ = 0.42, *SE* = 0.04). Overconfidence was more common for relative performance (*rp*_*overconfidence*_ = 0.52, *SE* = 0.01) than for absolute performance (*ap*_*overconfidence*_ = 0.39, *SE* = 0.02). This distribution is similar to that reported by [44].

Correlation results (please see S1 Table) show that absolute and relative overconfidence tended to co-occur in our data (*Pearson’s r* = 0.70, *p* = 0.00) and that they were negatively associated with participants’ performance on the quiz (oc: *r* = -0.58, *p* = 0.00; roc: *r* = -0.78, *p* = 0.00). Furthermore, absolute (*r* = 0.16, *p* = 0.02) and relative (*r* = 0.16, *p* = 0.02) overconfidence were more frequently observed in the *joy* treatment. In addition, absolute overconfidence was weakly negatively correlated with the *joy awareness* treatment (*r* = -0.12, *p* = 0.09). The overconfidence measures and some of the treatment groups were also weakly correlated with risk preferences, personality traits, and general self-efficacy. Furthermore, the age and employment status of the participants were not equally distributed across the treatment groups. We controlled for these potential confounds in the multivariate analyses below.

**Fig 2:**
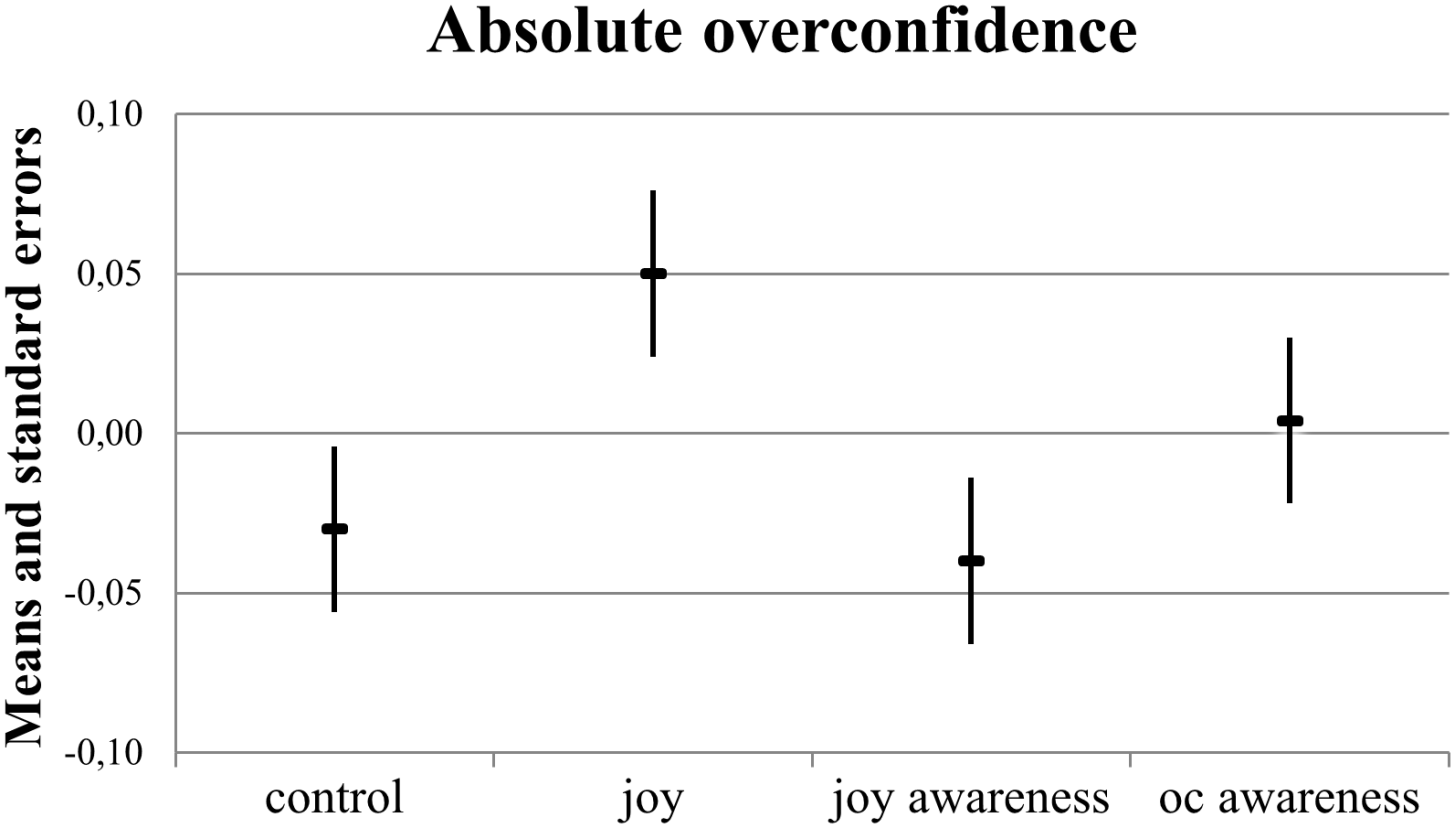
Absolute overconfidence across experimental groups, means, and standard errors (*N* = 213).

A descriptive comparison of absolute overconfidence across the experimental groups (Fig 2) shows that participants in the *joy* treatment group (*M* = 0.05, *SE* = 0.03) made overconfident judgments, whereas participants in the *control* group (*M* = -0.03, *SE* = 0.03) and the *joy awareness* group (*M* = -0.04, *SE* = 0.03) judged their performances as slightly underconfident. People in the *overconfidence awareness* group (*M* = 0.004, *SE* = 0.03) judged their absolute performance very accurately.

The difference between the *joy* treatment on absolute overconfidence and the *control* group was *t*(102) = 1.86, *p* = 0.07, *d* = 0.37. *Joy awareness* decreased absolute overconfidence compared with that measured for the *joy* group, with *t*(106) = 2.32, *p* = 0.02, *d* = 0.45. A one-sample, one-sided *t*-test shows that participants in the *joy* treatment were marginally overconfident in an absolute sense (*t*_*mean = 0*_ (52) = 1.56, *p*_*one-sided*_ = 0.06, *d* = 0.43).

**Fig 3:**
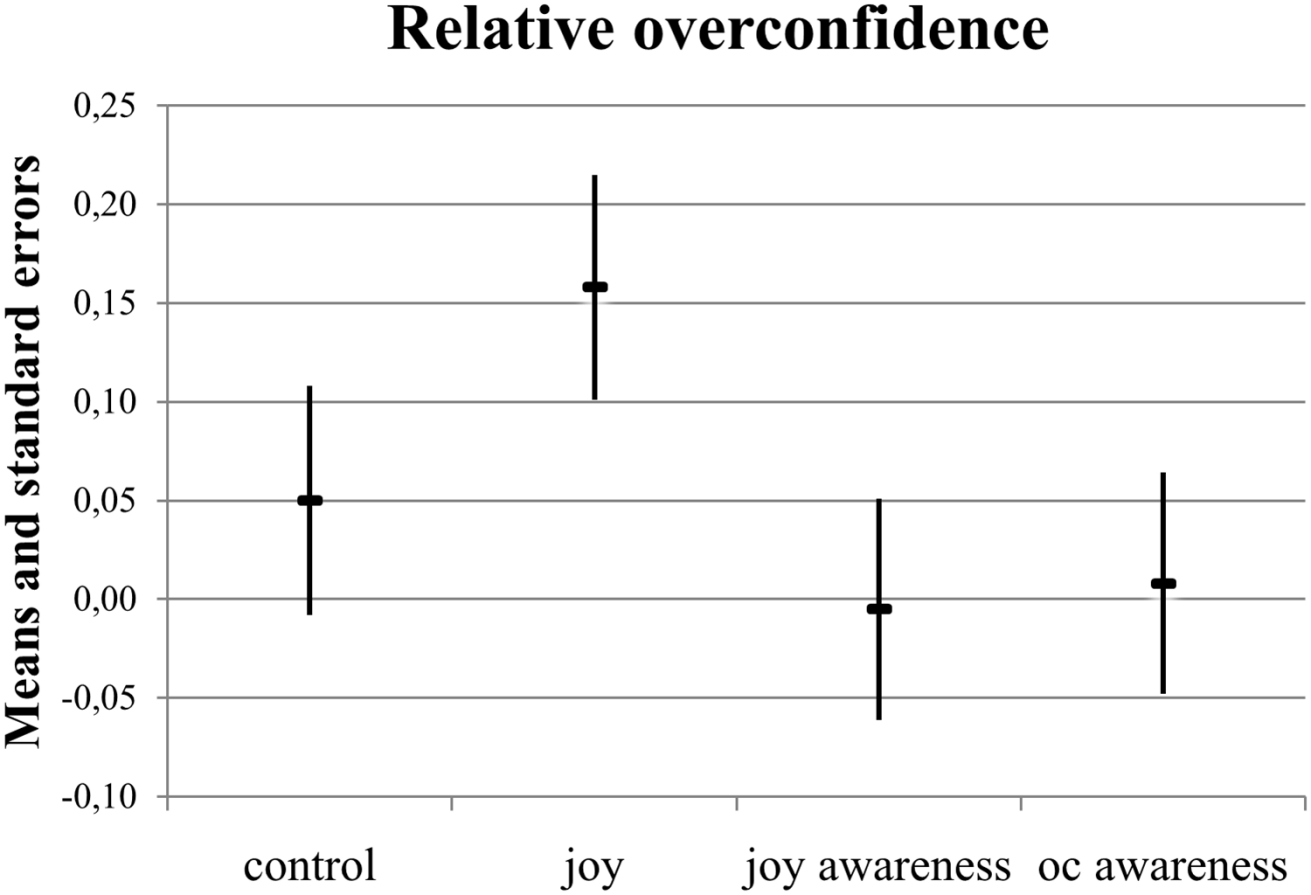
Relative overconfidence across experimental groups, means, and standard errors (*N* = 213).

Descriptive comparisons for relative overconfidence (Fig 3) indicate that the participants in the *joy* treatment group (*M* = 0.16, *SE* = 0.06) again showed the highest level of overconfidence, followed by the *control* group (*M* = 0.05, *SE* = 0.06). Both awareness groups (i.e., the *joy awareness* group [*M* = -0.01, *SE* = 0.06] and the *overconfidence awareness* group [*M* = 0.01, *SE* = 0.06]) showed well-calibrated confidence judgments about their relative performance.

The difference between the *joy* treatment on relative overconfidence and the *control* group was *t*(102) = 1.46, *p* = 0.15, *d* = 0.29. Compared with the *joy* treatment group, *joy awareness* decreased relative overconfidence, with *t*(106) = -2.31, *p* = 0.02, *d* = 0.40. The one-sample, one-sided *t*-test shows that the participants in the joy treatment group were on average overconfident in a relative sense (*t*_*mean = 0*_(52) = 2.87, *p*_*one-sided*_ = 0.003, *d* = 0.80).

As robustness tests of our findings, we ran analysis of variance (ANOVA) and four analyses of covariances (ANCOVA), i.e., ANOVA with control variables, using absolute and relative overconfidence as dependent variables and the experimental groups as the between-subjects factor (please see Table 3 for details about these robustness checks). In addition, all results are robust to the inclusion of the 13 initially excluded participants (*N* = 226). We also checked for gender differences by including interaction terms of gender and our treatment variables in analyses of variances. We did not find that our results for absolute (*F*[3, 205] = 0.88, *p* = 0.45) and relative (*F*[3, 205] = 1.71, *p* = 0.17) overconfidence differed with respect to gender. Finally, ordered logistic regressions and non-parametric Kruskal-Wallis H and Dunn’s post-hoc tests yielded the same results as our main model specifications outlined in Table 3.

**Table 3:** Robustness Checks (*N* = 213).

	Absolute overconfidence					Relative overconfidence				
	Model 1	Model 2	Model 3	Model 4	Model 5	Model 1	Model 2	Model 3	Model 4	Model 5
Control										
Joy treatment	*d* = 0.36*	*d* = 0.42*	*d* = 0.37^^^	*d* = 0.53**	*d* = 0.54**	*d* = 0.29	*d* = 0.27	*d* = 0.23	*d* = 0.46*	*d* = 0.54**
Joy awareness	*d* = 0.08	*d* = 0.02	*d* = 0.03	*d* = 0.09	*d* = 0.03	*d* = 0.13	*d* = 0.10	*d* = 0.11	*d* = 0.04	*d* = 0.02
OC awareness	*d* = 0.18	*d* = 0.18	*d* = 0.17	*d* = 0.40*	*d* = 0.31	*d* = 0.12	*d* = 0.09	*d* = 0.15	*d* = 0.06	*d* = 0.004
Joy treatment										
Joy awareness	*d* = 0.45**	*d* = 0.44*	*d* = 0.41*	*d* = 0.44*	*d* = 0.52**	*d* = 0.40*	*d* = 0.37^^^	*d* = 0.33^^^	*d* = 0.42*	*d* = 0.53**
OC awareness	*d* = 0.22	*d* = 0.24	*d* = 0.20	*d* = 0.12	*d* = 0.23	*d* = 0.40*	*d* = 0.36^^^	*d* = 0.37^^^	*d* = 0.39*	*d* = 0.54**
Joy awareness										
OC awareness	*d* = 0.27	*d* = 0.20	*d* = 0.20	*d* = 0.31	*d* = 0.28	*d* = 0.01	*d* = 0.005	*d* = 0.05	*d* = 0.02	*d* = 0.02
***Model diagnostics***	*F*(3, 209) = 2.38, *p* = 0.07	*F*(3, 202) = 2.17, *p* = 0.09	*F*(3, 195) = 1.78, *p* = 0.15	*F*(3, 194) = 3.16, *p* = 0.03	*F*(3, 208) = 3.51, *p* = 0.01	*F*(3, 209) = 1.95, *p* = 0.12	*F*(3, 202) = 1.59, *p* = 0.19	*F*(3, 195) = 1.48, *p* = 0.22	*F*(3, 194) = 2.38, *p* = 0.07	*F*(3, 208) = 3.87, *p* = 0.01
***partial η*** ^***2***^	0.03	0.03	0.03	0.05	0.05	0.03	0.02	0.02	0.04	0.05

*Note*. Cohen’s *d* was calculated using adjusted *M* and *SD*. The standard errors of Cohen’s *d*s are 0.2. Group differences with *p* < 0.01 are marked with **, *p* < 0.05 are marked with *; group differences with *p* < 0.10 are marked with ^. All analyses of variance were followed by Fisher’s least significant differences (LSD) post-hoc test.

Model 1: ANOVA for absolute and relative overconfidence between experimental groups.

Model 2: ANCOVA for absolute and relative overconfidence between experimental groups with demographic variables (age, age^2^, gender, employment status) and order of choice sets as control variables.

Model 3: like Model 2 but additionally controlling for risk preferences, personality traits, general self-efficacy, and optimism.

Model 4: like Model 3 but additionally controlling for participants’ performance as percentage of correctly answered quiz questions.

Model 5: ANCOVA for absolute and relative overconfidence between experimental groups with performance as a control variable.

Based on the observed effect sizes in Table 3, post-hoc power calculations show that we had 74% power to find the effect of *joy awareness* compared with *joy* on absolute overconfidence and 66% power to find the effect on relative overconfidence (one-sided tests with *p* = 0.05 using G*Power 3.1.7) [78].

As an additional robustness check, we compared subjects’ performance with the actual randomly chosen opponent during the experiment (please see Tables 4 and 5 for details). Similar to our main results, ROC was highest in the joy treatment and lowest in the joy awareness treatment. However, the differences between treatments were no longer statistically significant. Furthermore, we conducted nine additional random matches, calculated ROC for every individual, and repeated the statistical analyses. Because of the noise induced by the random matching, the average ROC value of groups fluctuated. However, we always observed ROC to be highest in the *joy* treatment. On average, across the ten random draws, the rank order of the four experimental treatments in terms of ROC was the same as in our main model reported in Table 3 and in line with our interpretation of the data.

**Table 4:** Means and standard errors for relative overconfidence based on randomly chosen opponent answers (*N* = 213).

	Relative overconfidence	
	*M*	*SE*
Control	-0.07	0.06
Joy treatment	0.004	0.06
Joy awareness treatment	-0.14	0.05
OC awareness treatment	-0.04	0.06

**Table 5:** Results for relative overconfidence based on randomly chosen opponent answers (*N* = 213).

	Relative overconfidence				
	Model 1	Model 2	Model 3	Model 4	Model 5
Control					
Joy treatment	*d* = 0.17	*d* = 0.07	*d* = 0.09	*d* = 0.13	*d* = 0.21
Joy awareness	*d* = 0.18	*d* = 0.16	*d* = 0.14	*d* = 0.08	*d* = 0.10
OC awareness	*d* = 0.07	*d* = 0.14	*d* = 0.07	*d* = 0.25	*d* = 0.15
Joy treatment					
Joy awareness	*d* = 0.36^	*d* = 0.06	*d* = 0.23	*d* = 0.21	*d* = 0.32^
OC awareness	*d* = 0.08	*d* = 0.22	*d* = 0.16	*d* = 0.12	*d* = 0.07
Joy awareness					
OC awareness	*d* = 0.24	*d* = 0.28	*d* = 0.07	*d* = 0.33^	*d* = 0.25
***Model diagnostics***	*F*(3, 209) = 1.08, *p* = 0.36	*F*(3, 22) = 1.01, *p* = 0.39	*F*(3, 195) = 0.80, *p* = 0.50	*F*(3, 194) = 1.13, *p* = 0.34	*F*(3, 208) = 1.11, *p* = 0.35
***partial η*** ^***2***^	0.02	0.02	0.01	0.02	0.02

*Note*. Cohen’s *d* was calculated using adjusted *M* and *SD*. Group differences with *p* < 0.01 are marked with **, *p* < 0.05 are marked with *; group differences with *p* < 0.10 are marked with ^. All analyses of variance were followed by Fisher’s least significant differences (LSD) post-hoc test.

Model 1: ANOVA for relative overconfidence between experimental groups.

Model 2: ANCOVA for relative overconfidence between experimental groups with demographic variables (age, age^2^, gender, employment status) and order of choice sets as control variables.

Model 3: like Model 2 but additionally controlling for risk preferences, personality traits, general self-efficacy, and optimism.

Model 4: like Model 3 but additionally controlling for participants’ performance as percentage of correctly answered quiz questions.

Model 5: ANCOVA for relative overconfidence between experimental groups with performance as a control variable.

## Discussion

Our study identifies joy as a relevant transient cause of absolute and relative overconfidence. Participants who received an unexpected gift (a small bag of gummy bears) (e.g., [62], [80]) at the beginning of the experiment were more overconfident in their judgments about their absolute and relative performance on a general knowledge quiz than were participants in the control group who did not receive such a gift. If a small present such as gummy bears can cause an increase in overconfidence, other, more intense joyful experiences may have even stronger and more enduring effects. For example, [91] found that a gift from an unknown source created longer lasting positive feelings than did an equivalent gift from a known source.

The finding that joy leads to overconfidence is consistent with affect-as-information theory [30, 60], which proposes that individuals use their pre-existing feelings as an informational cue for the decision at hand, leading to affect-congruent judgments. According to this logic, biased judgments and suboptimal decisions (e.g., overconfidence) occur if the reason for the positive affect (e.g., an unexpected gift, sunny weather, a funny movie) is not salient to the decision maker and is irrelevant to the judgment task. This biasing effect on judgments, which is caused by using affect as information, can be eliminated if the irrelevant source of the current affective state is salient (e.g., [32–34]).

Our results suggest a simple way of reducing overconfidence: If decision makers are aware of their current moods and consciously reflect on the (possibly irrelevant) reasons for their moods, they may be able to avoid misattributing their moods to their decisions and may consequently improve the quality of their judgments. If the indirect concept-priming mechanism [70, 74] had been applied instead of affect-as-information, our debiasing approach would not have been successful. Under concept-priming assumptions, subjects who are made aware of their affective misattribution would still use their positive affect for the retrieval of affect-related memories, leading to overconfidence. In contrast, the affect-as-information hypothesis is consistent with our results: When subjects were made aware that their good mood was caused by the unexpected gift, we observed well-calibrated judgments instead of overconfidence.

We do not know how long these improvements in judgment accuracy last. We also do not know what effects joy might have on overconfidence if it is induced by a relevant source. Under such circumstances, it may be possible that joy (or any other affective state) increases the quality of judgments. Future research could investigate how the relevance of an affective source influences individual’s overconfidence and decision-making quality. Furthermore, our findings are related to a general-knowledge task context in a tightly controlled experimental setting. It is an open question whether an intervention similar to our *joy awareness* treatment would be similarly effective in real-world situations. Testing whether our debiasing strategy against overconfidence also works in different settings would be a worthwhile area for future research. However, we note that the tightly controlled study design we chose was necessary to establish a clear causal effect of our intervention.

We did not expect to find that the participants in the control group were underconfident in an absolute sense and only slightly overconfident in a relative sense. In contrast, our pretest found that participants in the control group were on average overconfident to a degree comparable to that of the *joy* group in the main experiment reported above. The PANAS-X measures used in our pretest (but not in our main study) showed that the participants in the control group were in a positive resting mood. We suspect that the slightly different experimental environments contributed to this observed difference: The pretest in Germany was conducted in a behavioral laboratory featuring 24 computers that were located in a large room and were only separated by blinds between tables. In addition, the room had windows through which incoming sunlight may have affected participants’ affective states and their choices in the experiment. In contrast, the participants in the main experiment in the Netherlands were seated in small, closed booths without sunlight that did not allow the participants to see or hear any activities around them. We suspect (but did not measure) that being seated in such an isolated, closed booth had an undesirable and slightly negative influence on the subjects’ mood, which may in turn have influenced their confidence. Furthermore, the main experiment was conducted during the fall in the Netherlands, when the weather is typically grey and rainy, compared with summer in southern Germany, when the weather is generally warm and sunny.

An alternative explanation of our findings could be that subjects in the *joy* group ate the gummy bears before the experiment, whereas subjects in the *joy awareness* group did not. If this was the case, sugar consumption could have been the cause of overconfidence in the *joy* group, possibly mediated by elevated mood [92–95]. Our data do not report whether or when participants ate their gummy bears. It would be worthwhile for future research to investigate whether and how sugar consumption may be linked to overconfidence.

Because we did not observe overconfidence in the control group of our main experiment, we cannot determine whether the *overconfidence awareness* treatment is an effective debiasing strategy against overconfidence. We can only observe that mean judgments in the *overconfidence awareness* group were well calibrated on average. It may be promising to investigate in further studies whether and via which mechanism awareness of a bias alone can improve the quality of judgments and decisions.

The average effect sizes in our experiment comparing the *joy* and *joy awareness* groups (Avg(*d*) = 0.43 in Table 3) were slightly below our (over)optimistic expectation of *d* ≈ 0.5. Although this outcome led to a minor loss of achieved statistical power relative to our expectations, our study still fairs relatively well compared with most other experiments in psychology, which have an average power of only 0.5 [96].

We observed that our participants’ average judgment precision was lower for relative overconfidence than for absolute overconfidence. This outcome was reflected in standard errors that were twice as large on average for relative overconfidence than for absolute overconfidence (Figs 2 and 3) and a higher share of people who were relatively overconfident (Fig 1). A plausible explanation for this finding is the higher degree of uncertainty that people face when they compare themselves to others. Relative judgments of performance require both awareness about oneself and an estimate of the performance of others [46]. However, our suggested debiasing strategy against overconfidence was equally effective in the two scenarios. Specifically, our *joy awareness* group did not show evidence of absolute or relative overconfidence, whereas the *joy* group did.

Another important question that our study does not address is whether accurate, realistic judgments are always desirable. Optimistic self-delusion may be a positive causal factor that helps people cope with the challenges of life and persevere in the face of difficulty [97–100]. Indeed, some studies have reported positive associations between CEOs’ overconfidence and firms’ innovative performance [101, 102]. Similarly, more (over)confident entrepreneurs also self-report that they are more innovative than their less-confident colleagues [103]. The direction of causality is, however, unclear in all of these studies.

Despite the methodological challenges of identifying the effect of overconfidence on performance in field data, we acknowledge that overconfidence may indeed have partially positive effects: Theoretical work suggests that populations with a small share of overconfident individuals may actually have evolutionary advantages over populations with entirely unbiased perceptions [104].

Indeed, [40] and [105] have argued in two widely cited theoretical studies that optimistic self-delusion may even promote mental health, happiness, the ability to care for others, and the capacity for creative and productive work. Others have disagreed with this hypothesis and found evidence that overconfidence is related to problems with social interactions [106, 107] and depression [108, 109], contradicting the view that overconfidence is a positive aspect of mental health. Insofar as empirical studies find a positive relationship between joy, happiness, or subjective well-being and overconfidence [110, 111], our study suggests that the direction of causality may be opposite to what [40, 105] advocate: Rather than overconfidence promoting happiness, overconfidence may be the result of positive affect that the decision-maker has mistakenly misattributed as relevant information.

To the extent that overconfidence can be regarded as negative (such as in the examples we listed at the beginning), our results provide hope that effective, practical debiasing strategies exist that can help people make better judgments and decisions. This insight is particularly important because “the people who have the greatest influence on the lives of others are likely to be optimistic and overconfident and to take more risks than they realize” ([1], p. 256).

## Supporting Information

S1 Fig Absolute overconfidence across experimental groups, means, and standard errors in the pretest (*N* = 52). (TIF)

S2 Fig Absolute overconfidence across experimental groups, means, and standard errors in the pretest (*N* = 52). (TIF)

S1 Table Means (*M*), standard errors (*SE*), and correlation (*Pearson’s r*) results (*N* = 213). (DOCX)

S1 Text Pretest. (DOCX)

S2 Text Details about experimental design. (DOCX)
