# Supplementary figures and images for: Joy Leads to Overconfidence, and a Simple Countermeasure
Source: PLoS One. 2015 Dec 17;10(12):e0143263. doi: 10.1371/journal.pone.0143263 (PMC4683002; doi:10.1371/journal.pone.0143263)

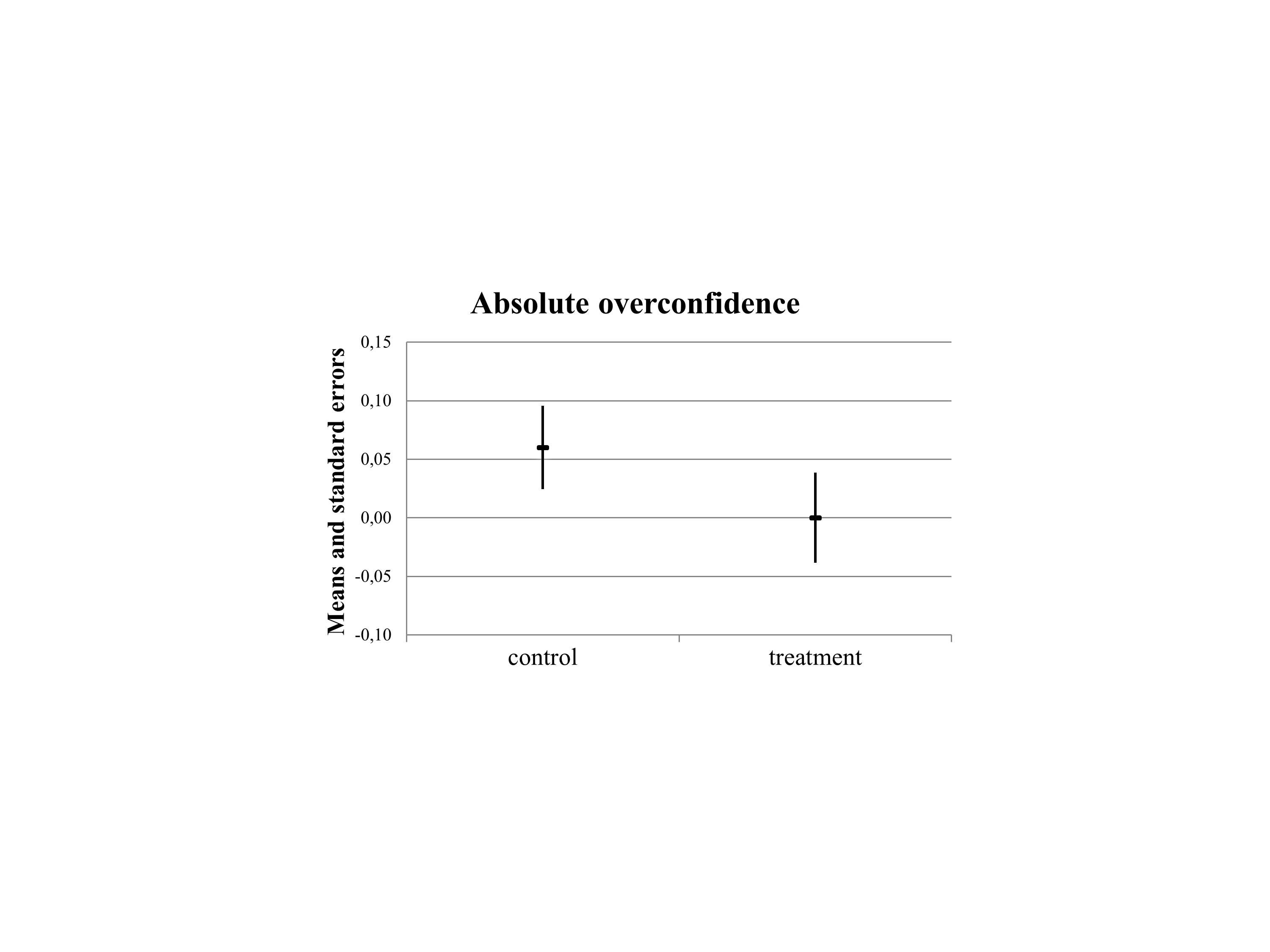

Supplement: S1 Fig — (TIF) [file pone.0143263.s001.TIF]

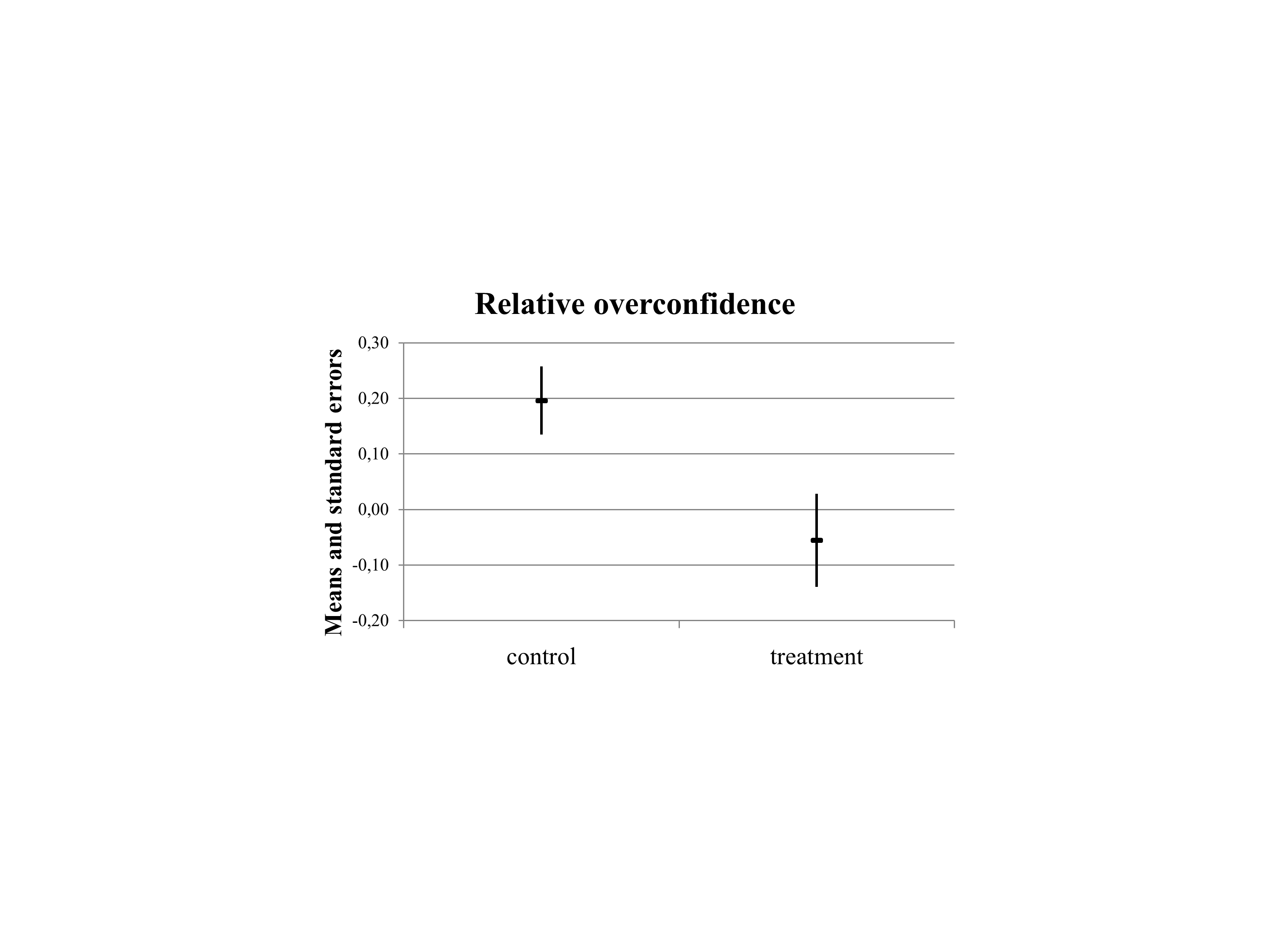

Supplement: S2 Fig — (TIF) [file pone.0143263.s002.TIF]
