# Supplementary material for: Joy Leads to Overconfidence, and a Simple Countermeasure
Source: PLoS One. 2015 Dec 17;10(12):e0143263. doi: 10.1371/journal.pone.0143263 (PMC4683002; doi:10.1371/journal.pone.0143263)
Supplement: S1 Table — (DOCX) [file pone.0143263.s003.docx]

|  | ***M*** | ***SE*** | **(1)** | **(2)** | **(3)** | **(4)** | **(5)** | **(6)** | **(7)** | **(8)** | **(9)** |
| --- | --- | --- | --- | --- | --- | --- | --- | --- | --- | --- | --- |
| **(1) Control** | 0.24 | (-) |  |  |  |  |  |  |  |  |  |
| **(2) Joy** | 0.25 | (-) | -0.32*** |  |  |  |  |  |  |  |  |
| **(3) Joy awareness** | 0.26 | (-) | -0.33*** | -0.34*** |  |  |  |  |  |  |  |
| **(4) OC awareness** | 0.25 | (-) | -0.33*** | -0.34*** | -0.34*** |  |  |  |  |  |  |
| **(5) OC** | -0.01 | 0.01 | -0.07 | 0.16** | -0.12^ | 0.03 |  |  |  |  |  |
| **(6) ROC** | 0.05 | 0.03 | 0 | 0.16** | -0.08 | -0.07 | 0.70*** |  |  |  |  |
| **(7) Performance** | 0.46 | 0.01 | -0.06 | -0.02 | 0.05 | 0.03 | -0.58*** | -0.78*** |  |  |  |
| **(8) Part** | 0.50 | (-) | -0.10 | 0.05 | -0.04 | 0.08 | -0.02 | -0.07 | 0.10 |  |  |
| **(9) Age** | 21.00 | 0.16 | -0.01 | 0.12^ | -0.07 | -0.04 | 0.10 | 0.14* | -0.01 | 0.01 |  |
| **(10) Male** | 0.55 | (-) | 0 | 0 | 0.02 | -0.01 | -0.04 | -0.11 | 0.17** | 0.06 | -0.03 |
| **(11) Employed** | 0.36 | (-) | 0.06 | -0.13* | 0.05 | 0.02 | -0.02 | -0.06 | -0.05 | -0.06 | -0.22** |
| **(12) Self-employed** | 0.04 | (-) | -0.06 | -0.07 | -0.02 | 0.15* | -0.01 | -0.07 | 0.11 | 0.07 | 0.12^ |
| **(13) Unemployed** | 0.24 | (-) | -0.14* | 0.08 | 0.06 | -0.01 | -0.07 | 0.05 | -0.01 | 0.02 | 0.09 |
| **(14) Risk** | 4.34 | 0.10 | -0.12^ | 0.11 | -0.01 | 0.03 | 0.01 | -0.02 | 0.06 | -0.01 | -0.03 |
| **(15) Extraversion** | (-) | (-) | -0.04 | 0.10 | 0.03 | -0.09 | 0.12^ | 0.06 | -0.02 | -0.01 | -0.09 |
| **(16) Emot. Stab.** | (-) | (-) | 0.10 | -0.07 | 0.05 | -0.08 | -0.03 | -0.12^ | 0.10 | 0.02 | -0.01 |
| **(17) Conscientious.** | (-) | (-) | 0.02 | -0.06 | 0 | 0.03 | -0.05 | -0.01 | -0.06 | 0.20** | 0.08 |
| **(18) Openness** | (-) | (-) | 0.04 | 0.09 | -0.02 | -0.04 | 0.02 | 0.02 | 0.01 | 0.02 | 0 |
| **(19) GSE** | 3.06 | 0.03 | 0.13* | 0.02 | 0.12^ | -0.26*** | -0.06 | -0.09 | 0.16* | -0.08 | -0.02 |
| **(20) Optimism** | 3.59 | 0.05 | -0.03 | 0.06 | 0.02 | -0.06 | 0.03 | -0.10 | 0.11 | -0.03 | -0.09 |

|  | **(10)** | **(11)** | **(12)** | **(13)** | **(14)** | **(15)** | **(16)** | **(17)** | **(18)** | **(19)** |
| --- | --- | --- | --- | --- | --- | --- | --- | --- | --- | --- |
| **(11) Employed** | 0.03 |  |  |  |  |  |  |  |  |  |
| **(12) Self-employed** | 0.10 | 0.14* |  |  |  |  |  |  |  |  |
| **(13) Unemployed** | 0.03 | -0.33*** | -0.12^ |  |  |  |  |  |  |  |
| **(14) Risk** | 0.15* | -0.01 | 0.08 | -0.04 |  |  |  |  |  |  |
| **(15) Extraversion** | -0.01 | -0.01 | 0.01 | 0.04 | 0.29*** | - |  |  |  |  |
| **(16) Emot. Stab.** | 0.23*** | 0.03 | -0.04 | -0.02 | 0.11^ | - | - |  |  |  |
| **(17) Conscientious.** | -0.11 | 0.05 | 0.09 | -0.11 | -0.05 | - | - | - |  |  |
| **(18) Openness** | 0.02 | 0.04 | 0.08 | -0.01 | 0.28*** | - | - | - | **-** |  |
| **(19) GSE** | 0.16* | 0.03 | -0.01 | -0.05 | 0.25*** | 0.17** | 0.27*** | 0.20** | 0.26*** |  |
| **(20) Optimism** | 0.14* | 0.11 | -0.01 | -0.12^ | 0.31*** | 0.35*** | 0.34*** | 0.04 | 0.08 | 0.28*** |

*Note.* Correlation coefficients are two-tailed, ^ *p* < 0.10, * *p* < 0.05, ** *p* < 0.01, *** p < 0.001.

Joy awareness = joy awareness group, OC awareness = overconfidence awareness group, OC = absolute overconfidence, ROC = relative overconfidence, Performance = percentage of correctly answered quiz questions, Part = sequence of measures (1 = absolute overconfidence first), Risk = risk preferences, Emot. Stab. = emotional stability, Conscientious. = conscientiousness, GSE = general self-efficacy. Personality traits are based on factor scores.
