# Supplementary material for: Joy Leads to Overconfidence, and a Simple Countermeasure
Source: PLoS One. 2015 Dec 17;10(12):e0143263. doi: 10.1371/journal.pone.0143263 (PMC4683002; doi:10.1371/journal.pone.0143263)
Supplement: S1 Text — (DOCX) [file pone.0143263.s004.docx]

The pretest was conducted at a large public university in Germany in 2010 with 52 students from various fields. The Ethics Committee from the University of Munich in Germany approved our pretest, and all participants provided written informed consent. The sample consisted of 22 males and 30 females with an average age of 25 years (*SE* = 0.79). The youngest and oldest participants were 18 and 45 years old. The pretest involved three different decision tasks, one of which was the current task on overconfidence. The other two tasks were unpublished measures of risk and ambiguity preferences. The participants completed the experiment with fixed (EUR 9, *N* = 22), low (up to EUR 11, *N* = 24), and high (possibility to win EUR 700, *N* = 6) financial stakes.

In each session, both a treatment (i.e., with a movie, comparable to the *joy awareness* group in our experiment, *N* = 27) and a control design (i.e., without a movie, comparable to the control group in our experiment, *N* = 25) were conducted with different participants. All participants completed 19 self-reported measures of their current affective state using three subscales (joy, fear and sadness) of the short version of the Positive and Negative Affect Schedule (PANAS-X) [112].

The treatment group watched a short, humorous movie clip (“When Harry met Sally” 1989, 3 min) [113] to induce positive affect ([79], [81]). Because people are generally in a positive resting mood (e.g., [31], [60]) and positive affect is often more difficult to induce than negative affect [79], we expected that the movie would induce only a small increase in joy among the participants in the treatment group. Prior to viewing the film clip, the participants were asked to become involved in the feelings suggested by the situation and to clear their minds of all thoughts, feelings and memories. Providing such explicit instructions increases the intensity of affect induction [81] and helps to make participants aware of the irrelevant cause of their affective state. After viewing the film clip, the participants were asked to complete a second affect measurement using the same PANAS-X subscales. All of the affect-related experimental elements in the treatment group very likely made the affect manipulation of the movie clip obvious to the participants. Hence, all participants were asked to reflect consciously upon their current affective state, but only the participants in the treatment group were confronted with a salient and non-relevant cause for their affect, i.e., the film clip.

The control group answered the PANAS-X subscales only once, at the beginning of the experiment. A mean comparison of self-reported joy using the relevant factor score from a Varimax-rotated principal component analysis of all 19 PANAS-X items shows that the control group was in a positive resting mood (*Mean* = 0.13, *SE* = 0.22) and that in the treatment group, the movie clip had the desired effect of increasing joy (*Mean* = 0.87, *SE* = 0.24), *t*(50)= 2.23, *p* = 0.03, *d* = 0.63. This procedure considers all available information, whereas average responses or factor scores, which are only based on the joy items, ignore mood differences among people arising from sadness or fear. However, all three possible measures of joy are almost perfectly correlated (*Pearson’s r*s > 0.95, *p* < 0.01).

**Results**

On average, participants answered 4.83 (*SE* = 0.23) out of 10 general knowledge questions correctly. The median performer answered five questions correctly. The best and the worst performances were eight questions (*N_8_* = 4) and one (*N_1_* = 1) question answered correctly, respectively. There was no significant difference between the treatment (*Mean* = 5, *SE* = 0.29) and the control (*Mean* = 4, *SE* = 0.36) groups in their performance (*t*(50) = -1.62, *p* = 0.11, *d* = 0.46) or their range of performance (test of homogeneity of variances, Levene’s *F*(50) = 0.49, *p* = 0.48, *d* = 0.46).

A descriptive comparison of overconfidence across the experimental groups (S1 and S2 Figs) shows that participants in the treatment group had better-calibrated judgments than participants in the control group. On average, judgments of absolute performance were exactly calibrated (*oc_treatment_* = 0.00, *SE* = 0.04) in the treatment group. The treatment group was also better calibrated in terms of the participants’ relative ability judgments (*roc_treatment_* = -0.06, *SE* = 0.08) compared with the control group. Participants in the control group were on average overconfident about their absolute (*oc_control_* = 0.06, *SE* = 0.04) and relative abilities (*roc_control_* = 0.20, *SE* = 0.06). The differences between the treatment and the control group are statistically distinguishable for relative (*t*[50] = 2.40, *p* = 0.02, *d* = 0.68) but not for absolute (*t*[50]=1.14, *p* = 0.26, *d* = 0.32) overconfidence. Furthermore, the control group was on average strongly overconfident in a relative sense (one-sample *t*-test, *t_mean=0_*[24] = 3.20, *p_one-sided_* = 0.002, *d* = 1.31) and less strongly overconfident in an absolute sense (one-sample *t*-test, *t_mean=0_*[24] = 1.69, *p_one-sided_* = 0.05, *d* = 0.69).

Correlation results show that relative and absolute overconfidence tended to co-occur in our data (*Pearson’s r* = 0.57, *p =* 0.01). Additional correlation results did not suggest any non-random distribution of relevant personal characteristics between the control group and the treatment group that could potentially bias the results.

**From the pretest toward the main experiment**

The results of our pretest suggest that the experimental manipulations we conducted had an effect on overconfidence. However, our experimental design in the pretest did not allow us to distinguish between two alternative interpretations of the results. The first explanation is that the higher level of joy in the treatment group actually improved judgment accuracy. This explanation would contradict affect-congruent findings and theories and the results of [30], who independently conducted a relatively similar mood induction in their overconfidence experiment. The second explanation is that awareness of the irrelevant source of joy in the treatment group (i.e., watching a movie clip and completing the PANAS-X scale before and after watching the clip) led to the decrease in overconfidence, which would be consistent with the affect-as-information theory ([31], [60]). Importantly, the study of [30] measured the self-reported moods of their participants *after* their overconfidence task, whereas our pretest measured moods *before* the task. Thus, our subjects were much more likely to realize before the overconfidence measurement that our purpose in showing them a movie clip was to manipulate their mood. This finding suggested that the awareness of the irrelevant source of joy and not the increased level of joy in the treatment group led to their improved judgment accuracy. We designed our main experiment to parse these two competing explanations and to explicitly test the role of mood salience for absolute and relative overconfidence.

**References**

112. Watson D, Clark LA. The PANAS-X: Manual for the Positive and Negative Affect Schedule - Expanded Form. Iowa City, IA: University of Iowa; 1994.

113. Hewig J, Hagemann D, Seifert J, Gollwitzer M, Naumann E, Bartussek D. A revised film set for the induction of basic emotions. Cog Emot. 2005; 19: 1095-1109.
