# Supplementary material for: Joy Leads to Overconfidence, and a Simple Countermeasure
Source: PLoS One. 2015 Dec 17;10(12):e0143263. doi: 10.1371/journal.pone.0143263 (PMC4683002; doi:10.1371/journal.pone.0143263)
Supplement: S2 Text — (DOCX) [file pone.0143263.s005.docx]

**X_3_ – Overconfidence awareness treatment**

Scientific studies have shown that decision making and behavior are largely influenced by various judgment errors and cognitive biases. One of the most notorious and widespread biases is overconfidence, i.e., the belief to be better than is actually warranted. In fact, most people are overconfident about their abilities and unreasonably optimistic about their future. Most people also claim to be above the median on almost any positive trait, although only half can actually be above the median.

We want you to be aware of the existence of overconfidence as a common bias in judgment and decision-making before starting with the experiment. Judging your own performance *accurately* will maximize your expected financial payoff in this experiment.

**O_1_ – Overconfidence measure**

You will now receive detailed instructions regarding the course of the experiment. It is crucial for the success of our study that you fully understand the instructions. After having read them, you will therefore have to answer 10 test questions to control whether you understood the instructions correctly. Please read the instructions carefully and do not hesitate to ask the experimenters in case you have any questions.

**Course of the experiment**

You will see a sequence of 10 quiz questions, for each of which you will have to choose 1 out of 5 possible answers. One other player will be randomly assigned to you and will have to solve exactly the same series of questions. In (the following) two parts, we will offer you the opportunity to choose a payoff mechanism. A payoff mechanism is a method that describes how your payoff will be determined. In both parts, you will have to choose between two options: cards and quiz.

**1. Cards**

For this mechanism, 20 playing cards will be shuffled. A certain number of these cards are green. You will draw one card from the stack. If the card is green, you will receive 6 €. If the card is not green, you will receive 0 €. By the time you have to decide for or against this payoff mechanism, you will know exactly how many of the cards are green.

**2. Quiz**

If you choose this mechanism, your payoff depends on your answers to the quiz questions. The more questions you have answered correctly, the higher is your chance of receiving a payoff of 6 €. There are two variants of the payoff mechanism “quiz”: own result and relative result.

a) **Own result**: One out of the 10 quiz questions will be drawn randomly. If you answered this question correctly, you will receive a payoff of 6 €. Otherwise, you will receive 0 €. With this payoff mechanism, your payoff will only depend on your own performance.

b) **Relative result**: If you answered more questions correctly than the player that has been assigned to you in the beginning and had to answer exactly the same questions, you will receive 6 €. If you answered fewer questions correctly, you will receive 0 €. In case of a draw, it will be randomly decided who will receive the 6 €.

In one part of the experiment, you will be able to choose between the payoff mechanisms

(1) cards and

(2) **quiz – own result**.

In another part of the experiment, you will be able to choose between the payoff mechanisms

(1) cards and

(2) **quiz – relative result**.

In both parts, one of your options will be to draw a card from a stack which may be green and which is a pure random mechanism. The other option will always be a payoff mechanism, which determines your payoff based on your result from answering the quiz questions. This means that, in any case, you should try to correctly answer as many questions as possible. The diagram below shows the course of the experiment schematically:

| Part 1 | Answer quiz questions | | |
| --- | --- | --- | --- |
| Part 2a | Choose a  payoff  mechanism | **1) Cards**  **or** | • One out of 20 cards is drawn  • Card is green: 6 €  • Card is not green: 0 € |
|  |  | **(2) Quiz –**  **own result** | • One quiz question is randomly drawn  • Correct answer: 6 €  • Wrong answer: 0 € |
| Part 2b | Choose a  payoff  mechanism | **(1) Cards**  **or** | • One out of 20 cards is drawn  • Card is green: 6 €  • Card is not green: 0 € |
|  |  | **(2) Quiz -**  **relative**  **result** | • Another player has been randomly  assigned to you  • You answered more questions correctly  than he/she: 6 €  • You answered fewer questions correctly  than he/she: 0 € |

If you have understood the course of the experiment, you may now start to answer the test questions you will see on the next computer screen. The sole aim of the test questions is to control whether you understood the instructions. They are not the quiz questions you will see in part 1 of the experiment!

**Test questions**

The following questions review if you understood the experimental instructions. You can only continue to the quiz questions once you have answered these ten questions correctly.

1. With the payoff mechanism **quiz – own result** I win 6 € if I answered the first quiz question right. (F)
2. With the payoff mechanism **quiz – own result** I win 6 €, if a randomly drawn quiz question I answered is correct. (C)
3. With the payoff mechanism **cards** my chances of winning improve the more questions I have answered correctly. (F)
4. With the payoff mechanism **quiz – relative result** I win 6 € if I answered more quiz questions correctly than the player that has been assigned to me in the beginning. (C)
5. With the payoff mechanism **cards** my chances of winning are the higher the more of the 20 cards are green. (C)
6. In one part of the experiment I have the option to be paid either according to my own result of the quiz or depending on if I played better than by teammate. (F)
7. In one part of the experiment I have to choose among several card stacks with a different amount of green cards. (F)
8. In both parts of the experiment I have to choose among the payoff mechanisms **cards** and **quiz**. (C)
9. Within this experiment it doesn’t matter which payoff mechanism I choose, as my payoff depends anyway on chance. (F)
10. With the payoff mechanism **quiz – relative result** it is possible that the player that has been assigned to me had to respond to easier questions. (F)

**Quiz questions**

Please answer the following 10 quiz questions by choosing 1 out of 5 possible answers. There is only one correct answer to every question.

| 1. | Earth equator is around … 000 km long. | ***40*** | 24 | 36 | 52 | 14 |
| --- | --- | --- | --- | --- | --- | --- |
| 2. | Solar system consists of… known planets (recent counting). | 13 | ***8*** | 12 | 9 | 17 |
| 3. | First Tour de France took place in year | 1898 | 1915 | ***1903*** | 1814 | 1938 |
| 4. | Which triple of notes contains C major? | A-C-B | D-F-A | C-D-G | F-C-D | ***C-E-G*** |
| 5. | Ludwig van Beethoven wrote… symphonies. | 15 | ***9*** | 41 | 13 | 104 |
| 6. | The frequency of voltage in central Europe is … Hz. | 220 | 110 | ***50*** | 66 | 85 |
| 7. | Human cell consists of… chromosomes. | 32 | 58 | ***46*** | 38 | 23 |
| 8. | Human body has… sense organs. | 4 | ***5*** | 6 | 7 | 8 |
| 9. | Sum of angles in a triangle is … degrees. | 360 | 380 | 60 | 90 | ***180*** |
| 10. | “Lord of the Rings” is based on a book by… | ***Tolkien*** | Tolstoy | Trotzki | Thomas | Trevier |

Please choose in this payoff mechanism between *quiz-own result* and cards:

|  | ***Quiz-own result*** | ***cards*** |
| --- | --- | --- |
| If there are 1 green and 19 white cards in the stack, and I have the choice between quiz-own results and cards, I choose | O | O |
| If there are 3 green and 17 white cards in the stack, and I have the choice between quiz-own results and cards, I choose | O | O |
| If there are 5 green and 15 white cards in the stack, and I have the choice between quiz-own results and cards, I choose | O | O |
| If there are 7 green and 13 white cards in the stack, and I have the choice between quiz-own results and cards, I choose | O | O |
| If there are 9 green and 11 white cards in the stack, and I have the choice between quiz-own results and cards, I choose | O | O |
| If there are 11 green and 9 white cards in the stack, and I have the choice between quiz-own results and cards, I choose | O | O |
| If there are 13 green and 7 white cards in the stack, and I have the choice between quiz-own results and cards, I choose | O | O |
| If there are 15 green and 5 white cards in the stack, and I have the choice between quiz-own results and cards, I choose | O | O |
| If there are 17 green and 3 white cards in the stack, and I have the choice between quiz-own results and cards, I choose | O | O |
| If there are 19 green and 1 white cards in the stack, and I have the choice between quiz-own results and cards, I choose | O | O |

Please choose in this payoff mechanism between *quiz-relative result* and cards:

|  | ***Quiz-relative result*** | ***cards*** |
| --- | --- | --- |
| If there are 1 green and 19 white cards in the stack, and I have the choice between quiz-relative results and cards, I choose | O | O |
| If there are 3 green and 17 white cards in the stack, and I have the choice between quiz- relative results and cards, I choose | O | O |
| If there are 5 green and 15 white cards in the stack, and I have the choice between quiz- relative results and cards, I choose | O | O |
| If there are 7 green and 13 white cards in the stack, and I have the choice between quiz- relative results and cards, I choose | O | O |
| If there are 9 green and 11 white cards in the stack, and I have the choice between quiz- relative results and cards, I choose | O | O |
| If there are 11 green and 9 white cards in the stack, and I have the choice between quiz- relative results and cards, I choose | O | O |
| If there are 13 green and 7 white cards in the stack, and I have the choice between quiz- relative results and cards, I choose | O | O |
| If there are 15 green and 5 white cards in the stack, and I have the choice between quiz- relative results and cards, I choose | O | O |
| If there are 17 green and 3 white cards in the stack, and I have the choice between quiz- relative results and cards, I choose | O | O |
| If there are 19 green and 1 white cards in the stack, and I have the choice between quiz- relative results and cards, I choose | O | O |
